# A Cross-Cultural Examination of Common Root Canal Mistakes in Spanish Dental School

**DOI:** 10.1155/ijod/8863750

**Published:** 2025-07-10

**Authors:** Fátima Martín-Hernán, Rosa María Jiménez, Patricia Alejandra Lara-López, Rosa María Vilariño-Rodríguez, Juan Manuel Aragoneses-Lamas, Rosa Rojo

**Affiliations:** ^1^Faculty of Dentistry, Alfonso X El Sabio University, Villanueva de la Cañada 28691, Community of Madrid, Spain; ^2^Faculty of Dentistry, Dental Clinic, Alfonso X El Sabio University, Madrid 28037, Community of Madrid, Spain

**Keywords:** clinical training, educational programs, endodontic teaching, endodontic treatments, quality, students

## Abstract

**Introduction:** Endodontic treatments require a high level of manual skill and operative independence, which is why preclinical training and the development of appropriate educational programs are vital. This study aimed to evaluate the quality of endodontic treatments and the influence of nationality on undergraduate academic performance at a Spanish dental school with a large proportion of international students.

**Material and Methods:** A cross-sectional study was carried out on students of the Dental Therapeutic Pathology II subject at the Faculty of Dentistry in the 2022–2023 academic year. Two independent evaluators evaluated the quality of the treatments, and errors made during the treatment were identified. The chi-square test assessed the association between the errors committed in root canal treatment and nationality.

**Results:** A total of 319 students were evaluated. The most frequent nationality was French (57.4%), followed by Italian (22.9%), Spanish (16.0%), and others (3.8%). Treatment of multiple ducts was the most performed (75.86%). The French carried out the highest number of treatments (73.22%). The most frequent error was identified in the design of the camera aperture. There were no statistically significant differences between the number of errors and nationality.

**Conclusions:** A growing internationalization of students has been identified. Despite cultural and linguistic differences, a common tendency to make errors in the design of openings in endodontic treatments was observed in all groups. French students stood out with superior academic performance.

## 1. Introduction

Endodontics constitutes one of the great branches of dentistry. It is estimated that around the world, 41,000 teeth undergo endodontic treatment every day, and every year, 15 million people undergo endodontic treatment [1].

Compared to other specialties, endodontics requires a high level of manual skill and operative independence [2]. Preclinical training plays a crucial role in the learning and training of students so that they can become familiar with the procedures and techniques necessary to treat the patient properly[3]. Therefore, endodontic teaching programs must include training in tactile development and knowledge of dental anatomy, both external and internal, of all phases of endodontic treatment [4–8].

The European Society of Endodontology (ESE) proposed curricular guidelines that would serve as a reference in the clinical training of students and guide dental schools in designing of their educational programs in endodontics [9]. Despite significant technological advances and the publication of quality guidelines, very disappointing technical standards have continued to be found in the last 10 years, which is why these guides have been updated [10].

In this context, it is important to consider a series of factors that influence the student's learning process. These factors include the stress associated with the workload and academic expectations, the clinical experience and pedagogical skills of the teachers, the quality of the relationship between the student and the teacher, the ability to apply theory in practical situations, participation in extracurricular activities that complement their training, the student's self-esteem and their level of dedication. These elements, together, shape each student's learning experience and can influence their ability to achieve desired standards in endodontics [11–14].

Additionally, it is relevant to highlight that more and more students choose to complete their higher education in foreign countries, which implies learning a different language. This global trend towards the internationalization of higher education poses additional challenges in teaching endodontics, as students must face a new academic environment and the language barrier. This multicultural and linguistic experience can influence students' adaptation and learning capacity [15–17].

An evaluation of endodontic training programs is required to address not only traditional factors but also consider new dynamics, such as the increasing internationalization of students. To date, no studies have been identified in the field of endodontics that focus on this perspective. Therefore, the objective of this study was to evaluate the quality of endodontic treatments and the influence of nationality on undergraduate academic performance at a Spanish dental school with a large proportion of international students.

## 2. Methods

### 2.1. Study Design

The study was carried out following the Strenthening the Reporting of Observational Studies in Epidemiology (STROBE) recommendations [18]. This cross-sectional study was carried out with students of the dental therapeutic pathology II subject at the faculty of dentistry. Anonymized data from students from the 2022–2023 academic year was used. They were provided by the head of studies of the degree in dentistry. The ethics committee was approved on January 25, 2021 with the number 25/01/21 was granted by the Ethical Committee of Federico Henriquez y Carvajal University in accordance with the Declaration of Helsinki (Adopted by the 18^th^ WMA General Assembly, Helsinki, Finland, June 1964 and amended by the 64th WMA General Assembly, Fortaleza, Brazil, October 2013).

As a sample, information was collected from all students enrolled in the dental therapeutic pathology II subject of the fourth year of the degree in dentistry.

For root canal treatment, students worked on natural teeth mounted on acrylic typodonts (FRASACO type AG-3 Z) and on phantoms (BADER) to simulate clinical reality. All teeth were instrumented with ISO stainless steel K-Flexofile manual files (Dentsply Maillefer; Dentsply Sirona Iberia; Valencia) and filled using the cold lateral condensation technique with ISO-compatible gutta-percha and auxiliary tips (Dentsply Sirona Maillefer; Dentsply Sirona Iberia; Valencia).

### 2.2. Criteria for Selection

The inclusion criteria were primary root canal treatments in uniradicular, biradicular, and multiradicular permanent teeth performed manually by university students enrolled in the fourth year of the dental degree. Root canals should have radiographs after obturation showing the entire length of the root. The exclusion criteria were all those treatments for which there were superimposed fillings or anatomical structures in radiographs after obturation.

### 2.3. Procedure for Root Canal Treatment

Five periapical radiographs were required for all root canal treatment cases: diagnostic radiograph, conductometric radiograph for working length determination, conductometric radiograph, condensation radiograph, and final radiograph after tooth reconstruction.

Periapical radiographs were taken with phosphor plates, processed with the PSPIX digital scanner (Acteon Group Ltd, Norway), and evaluated by the professors using Sopro Imaging 2.41 software (ISO file, 1.1 GB), the user interface of the Sopix PSPIX (Acteon Group Ltd, Norway) system.

The steps for performing the root canals were (1) preparation of the access cavity, (2) determination of the working length, (3) cleaning and hand instrumentation with ISO steel files (Dentsply Sirona), (4) obturation of the canal with 2% ISO gutta-percha points (Dentsply Sirona) and Top-Seal cement (Dentsply Sirona).

All teeth were treated with conventional ISO files, prepared using the step back technique, and obturated using the cold lateral condensation technique.

The step back technique was performed with ISO stainless steel hand K-files (Dentsply Maillefer Switzerland). The coronal expansion was initially performed with Gate Glidden drills size 02, 03, and 04 (Dentsply Maillefer, Switzerland). The K files were used to clean and shape the canal in the following sequence: #10, #15, #20, #25, #30, #35, #40, #45, #50, and #60. The working length was considered acceptable if it was within 0.5–1 mm of the radiographic apex as determined by periapical radiography.

The canals were irrigated with 5% sodium hypochlorite (Prevest DentPro Limited, Jammu, India) and obturated with ISO tips (Dentsply Maillefer, Switzerland) and Top Seal resin sealer (Dentsply Maillefer, Switzerland).

### 2.4. Evaluation of the Quality of Treatments and Variables

The quality of root canal treatments was evaluated, considering the ESE criteria [10]. The prepared root canal must be filled and maintain the original shape of the root canal; there must be no visible spaces between the filling material and the canal wall, nor in the visible space beyond the final point of root filling.

The canal filling was considered insufficient (infra obturation) when the root canal filling material was more than 1 mm from the radiographic apex. Excessive filling (overfilling) when the root canal filling material exceeded the radiographic apex. Any voids, inhomogeneities, and uniform narrowing were considered inadequate lateral condensation.

The following variables were collected: age, sex, status as a repeat student, nationality, number of root canal treatments performed (single-root, biradicular, and multi-root), and number and types of errors made.

The detected errors were classified into three categories. Those committed during the opening of the pulp chamber (such as incorrect design in the chamber opening, incorrect removal of the chamber roof, perforation, false pathways, or omitted canals), instrumentation (such as apical transports, lack of stop apical, steps, instrument fracture or canal blockages), and condensation (such as undersaturation, oversaturation, underextension, or overextension).

The evaluators of root canal treatment quality were two professors of the subject, specialists in endodontics and with at least 8 years of teaching and professional experience. They were precalibrated with the evaluation of 90 radiographs (30 for single-root canal treatments, 30 for bi-root canal treatments, and 30 for multi-root treatments) from the previous academic year and performed the assessment independently. In case of doubt, the subject coordinator intervened to decide.

### 2.5. Statistical Analysis

Intra- and inter-examiner reproducibility was assessed using the Cohen's kappa statistic. For continuous quantitative variables, the mean and standard deviation were presented. For the discrete quantitative variables and the qualitative variables, the frequencies and percentages. The Shapiro–Wilk test was performed to assess the normality of the variables. The Kruskal–Wallis test was performed to compare the number of treatments performed and the nationality. The chi-square test assessed the association between the errors committed in root canal treatment and nationality. The statistical program SPSS version 23.0 software (IBM, New York, USA) was used. All test results with a *p*-value < 0.05 were statistically significant.

## 3. Results

Three hundred nineteen students of the fourth-year dentistry degree course of dental therapeutic pathology II were evaluated, with a mean age of 26.08 years (SD = 3.45 years), 45.1% women, and 3.4% repeat students. The intra-examiner agreement was *k* = 0.9 and inter-examiner agreement was *k* = 0.8. The most frequent nationality among the students was French (57.4%), followed by Italian (22.9%), Spanish (16.0%), and others (3.8%) (Table 1).

### 3.1. Root Canal Treatments Performed During the Course

The number of root canal treatments completed by the students was 202 for single-root canals, 198 for bi-root canals, and 242 for multi-root canals. Multi-root canal treatment was the most performed (75.86%). There were statistically significant differences between the number of single root canal treatments performed and nationality (*p*=0.048), with the French performing the most treatments (73.22%) and the Spanish performing the least (41.18%) (Table 2).

### 3.2. Mistakes Made in Root Canal Treatments

The total number of errors made in single root canal treatments was 202 (63.32%); in biradicular canal treatments, it was 198 (62.07%), and in multi-root canal treatments, it was 242 (75.86%). No statistically significant differences existed between the number of errors and nationality (Table 3).

Table 4 shows the types of errors identified in each root canal treatment. It was in single-root canal treatments where the students presented fewer errors (17.24%) and in multi-root treatments where the most (0.94%). Regarding the types of errors, the most frequent treatment of single root canals occurred in the opening phase of the pulp chamber (13.48%). There were statistically significant differences between the errors made and the nationality (*p*=0.028).

The most frequent errors in biradicular canal treatment occurred in the phase of opening the pulp chamsber in combination with instrumentation (18.18%), in the condensation phase (12.85%), and all phases of root canal treatment (10.34%). There were statistically significant differences between the errors made and the nationality (*p*=0.034).

The most frequent errors in multi-root canal treatment occurred in the phase of opening the pulp chamber in combination with condensation (21.00%) and in all phases of root canal treatment (20.38%).

## 4. Discussion

While learning the manual endodontic technique in undergraduate dental students, errors have mainly been identified in the opening of the pulp chamber. More than 75% of the students have international origins, which could have influenced their academic performance.

Preclinical training in endodontics is essential to develop manual skills. In schools in the United Kingdom [19], Italy [3] or Spain [20], education in endodontics has been evaluated in undergraduate students by adapting the questionnaire developed by Qualtrough and Dummer [21]. This questionnaire evaluates aspects of the teaching provided in endodontics (both in preclinical and clinical), the subjects covered, the teaching resources, the teaching calendar, the allocation of teaching time, the training of teaching staff, the staff ratio (teacher/student ratio), as well as the materials and recommended instruments. All these factors are influential in academic performance.

In some universities, a minimum number of root canal treatments is not required, or they can fluctuate between 5–30 endodontics per student. The university of the present study, the minimum criteria in the subject involve performing a root canal in each type of root canal: single-root, bi-root, and multi-root. The most frequently performed treatment was multi-root (75.9%) and the least single-root (63.3%). In comparison with other European universities, such as Italian ones, the percentage of multi-root treatments is similar (79.2%), and in the case of single-root treatments much lower (91.7%) [3]. However, at university of the present study, biradicular and multiroot treatments are performed more frequently. However, biradicular and multi-root treatments at university of the present study, are performed more frequently. Due to the anatomical complexity of the molars, the satisfactory completion of these teeth gives the student better training and greater self-esteem [13].

The lowest number of errors in endodontic treatments was in single-rooted teeth (56.1%). As the number of canals increases, errors are observed in two-root treatments in 61.1% of the teeth and multi-root treatments in 75.9% of the teeth. This is due to the tooth's anatomy, a single root canal, and shape (wider and straighter), favoring operability [22].

The design of the pulp chamber opening was the most frequently occurring error, followed by condensation. Various studies have been carried out to evaluate the quality of root canal treatments in undergraduate dental students. In all of them, it is observed that it is in the condensation phase where the most errors occur (such as underfilling, overfilling, and the presence of spaces inside the duct due to lack of lateral condensation) [23, 24 ]. This is usually more common in multirooted teeth, as occurred at university of the present study. However, in the study by Tavares et al. [4] in Brazil, according to the student's perception, their most significant difficulties are not found in the instrumentation or obturation but in the radiographic evaluation. For this reason, the teaching programs of some universities incorporate the combined use of the ray technique and the apex locator or the apex locator exclusively for determining the working length [3]. In this study, students only use radiographic evaluation techniques.

Although this study evaluated the results of root canal treatment performed with manual instruments, students are also trained using procedures based on rotary instrumentation, although not with reciprocating rotary systems during the course. This occurs in most Spanish universities [20] and other European universities [3].

We find that factors that influence student learning are related to the teacher. In 65% of dental schools in Spain, students are supervised by full-time professors specializing in endodontics [20]. In this case, the teachers had at least 8 years of experience in endodontic treatments.

On the other hand, and less studied in the field of teaching in dentistry, is the language barrier and how it can influence teaching. In this study, teaching is taught in Spanish and most of the students are foreign: 57.4% French, 22.9% Italian, and 3.8% from other countries. French students had better academic results. Some studies suggest that international students face various challenges in their academic endeavors, including cultural and linguistic barriers, integration problems, and the need for additional support from institutions and professors [25–27]. Bijsmans et al. [28] explored the impact of internationalization on students and the success of their studies, discovering that there are positive and negative effects depending on the number of nationalities present. The study by Rienties et al. [29] revealed that international students of Western ethnic origin, as in this study, obtain good results in both academic and social integration. However, international students have yet to be studied as a mobile population in today's globalized world. Overall, international students' language proficiency and cultural background can influence their academic performance and highlight the need for support and interventions to improve their educational experiences. The results at the present study, highlight the need to promote an inclusive and diverse educational environment, offer specific support to students of various nationalities to improve their skills in technical areas, and evidence a need to explore this field further.

Regarding the limitations of this study, they include lack of randomization in the allocation of dental pieces and the potential bias due to the level of anatomical difficulty. In addition, strategies have been proposed to reduce errors, especially in the coronal opening phase, such as reinforcing three-dimensional anatomical training through digital models and virtual simulators.

In conclusion, this study in a university institution in Spain has identified a growing internationalization of students, with a notable presence of French, Italian, and Spanish nationalities. Despite cultural and linguistic differences, a common tendency to make errors in opening design in endodontic treatments was observed in all groups. Furthermore, it was observed that French students stood out with superior academic performance compared to other nationalities.

## Figures and Tables

**Table 1:** Demographic characteristics of the students.

Variable	French (*n* = 183)	Spanish (*n* = 51)	Italian (*n* = 73)	Others (*n* = 12)	Total (*N* = 319)	*p*-Value
Age (mean [SD])	25.63 (1.87)	27.23 (6.39)	26.36 (3.58)	26.42 (2.70)	26.08 (3.45)	0.712
Sex (women [%])	81 (44.26)	25 (49.02)	30 (41.10)	8 (66.67)	144 (45.1)	0.377
Repeater student (yes [%])	7 (3.83)	1 (1.96)	3 (4.11)	0 (0.00)	11 (3.4)	0.815
Total (%)	183 (57.4)	51 (16.0)	73 (22.9)	12 (3.8)	—	—

*Note:* Demographic characteristics of the students. Kuskall–Wallis test to assess age and nationality. Chi-square test to assess the association between nationality with age and sex. *p*-Values < 0.05 are statistically significant.

**Table 2:** Number of root canal treatments performed according to nationality.

Type of treatment	French (*n* = 183)	Spanish (*n* = 51)	Italian (*n* = 73)	Others (*n* = 12)	Total (*n* = 319)	*p*-Value
Single root canals						
0 (*n*, %)	0 (0.00)	1 (4.55)	0 (0.00)	0 (0.00)	—	—
1 (*n*, %)	134 (100.00)	21 (95.45)	39 (100.00)	8 (100.00)	—	—
Total performed (%, per group)	134 (73.22)	21 (41.18)	39 (53.42)	8 (66.67)	202 (63.32)	0.042
Biradicular root canals						
0 (*n*, %)	2 (1.49)	0 (0.00)	1 (2.56)	2 (25.00)	—	—
1 (*n*, %)	132 (98.51)	22 (100.00)	38 (97.44)	6 (75.00)	—	—
Total performed (%, per group)	132 (72.13)	22 (43.14)	38 (52.05)	6 (50.00)	198 (62.07)	≤0.001
Multiple root canals						
0 (*n*, %)	4 (2.96)	0 (0.00)	2 (5.13)	1 (12.50)	—	—
1 (*n*, %)	100 (74.07)	16 (72.73)	30 (76.92)	6 (75.00)	—	—
2 (*n*, %)	31 (22.96)	6 (27.27)	7 (17.95)	1 (12.50)	—	—
Total performed (%, per group)	162 (88.52)	28 (54.90)	44 (60.27)	8 (66.67)	242 (75.86)	0.484

*Note:* Frequencies represent the number (%) of students who performed the corresponding type of root canal. Kruskal–Wallis test was used to compare the number of root canal treatments performed across nationalities. *p*-values < 0.05 were considered statistically significant.

**Table 3:** Number of errors made in root canal treatments performed according to nationality.

Type of treatment	French (*n* = 183)	Spanish (*n* = 51)	Italian (*n* = 73)	Others (*n* = 12)	Total (*N* = 319)	*p*-Value
Single root canals with errors						
0 (*n*, %)	14 (10.45)	5 (22.73)	5 (12.82)	0 (0.00)	—	—
1 (*n*, %)	120 (89.55)	17 (77.27)	34 (87.18)	8 (100.00)	—	—
Total with errors (%, per group)	120 (65.57)	17 (33.33)	34 (46.58)	8 (66.67)	179 (56.11)	0.279
Biradicular root canals with errors						
0 (*n*, %)	2 (1.53)	0 (0.00)	0 (0.00)	0 (0.00)	—	—
1 (*n*, %)	129 (98.47)	22 (100.00)	38 (100.00)	6 (100.00)	—	—
Total with errors (%, per group)	129 (70.49)	22 (43.14)	38 (52.05)	6 (50.00)	195 (61.13)	0.798
Multiple root canals with errors						
0 (*n*, %)	1 (0.76)	1 (4.55)	0 (0.00)	1 (12.50)	—	—
1 (*n*, %)	99 (75.00)	15 (68.18)	30 (81.08)	6 (75.00)	—	—
2 (*n*, %)	32 (24.24)	6 (27.27)	7 (18.92)	1 (12.50)	—	—
Total with errors by nationality (%, per group)	163 (89.07)	27 (52.94)	44 (60.27)	8 (66.67)	242 (75.86)	0.586

*Note:* Frequencies represent the number (%) of students who made errors during the corresponding root canal treatments. Kruskal–Wallis test was used to assess differences in the number of errors by nationality. *p*-values < 0.05 were considered statistically significant.

**Table 4:** Types of errors committed in root canal treatments performed according to nationality.

Type of error	French (*n* = 183)	Spanish (*n* = 51)	Italian (*n* = 73)	Others (*n* = 12)	Total (*n* = 319)	*p*-Value
Type of single root canals performed with errors						0.028
There is no defect (frequency [%])	33 (24.63)	9 (40.91)	13 (34.21)	0 (0.00)	55 (17.24)	—
Opening of the pulp chamber (frequency [%])	27 (20.15)	7 (31.82)	5 (13.16)	4 (50.00)	43 (13.48)	—
Instrumentation (frequency [%])	6 (4.48)	0 (0.00)	6 (15.79)	2 (25.00)	14 (4.39)	—
Condensation (frequency [%])	20 (14.93)	0 (0.00)	6 (15.79)	1 (12.50)	27 (8.46)	—
Opening of the pulp chamber and instrumentation (frequency [%])	8 (5.97)	0 (0.00)	0 (0.00)	0 (0.00)	8 (2.51)	—
Opening of the pulp chamber and condensation (frequency [%])	21 (15.67)	3 (13.64)	4 (10.53)	0 (0.00)	28 (8.78)	—
Instrumentation and condensation (frequency [%])	5 (3.73)	1 (4.55)	3 (7.89)	1 (12.50)	10 (3.13)	—
Opening of the pulp chamber, instrumentation, and condensation (frequency [%])	14 (10.45)	2 (9.09)	1 (2.63)	0 (0.00)	17 (5.33)	—
Type of biradicular root canals performed with errors						0.034
There is no defect (frequency [%])	6 (4.58)	0 (0.00)	1 (2.63)	1 (14.29)	8 (2.51)	—
Opening of the pulp chamber (frequency [%])	14 (10.69)	1 (4.55)	8 (21.05)	1 (14.29)	24 (7.52)	—
Instrumentation (frequency [%])	7 (5.34)	4 (18.18)	2 (5.26)	3 (42.86)	16 (5.02)	—
Condensation (frequency [%])	30 (22.90)	5 (22.73)	6 (15.79)	0 (0.00)	41 (12.85)	—
Opening of the pulp chamber and instrumentation (frequency [%])	2 (1.53)	2 (9.09)	0 (0.00)	0 (0.00)	4 (1.25)	—
Opening of the pulp chamber and condensation (frequency [%])	43 (32.82)	5 (22.73)	9 (23.68)	1 (14.29)	58 (18.18)	—
Instrumentation and condensation (frequency [%])	8 (6.11)	2 (9.09)	4 (10.53)	0 (0.00)	14 (4.39)	—
Opening of the pulp chamber, instrumentation, and condensation (frequency [%])	21 (16.03)	3 (13.64)	8 (21.05)	1 (14.29)	33 (10.34)	—
Type of multiple root canals performed with errors						0.281
There is no defect (frequency [%])	2 (1.53)	1 (4.55)	0 (0.00)	0 (0.00)	3 (0.94)	—
Opening of the pulp chamber (frequency [%])	6 (4.58)	0 (0.00)	3 (8.11)	0 (0.00)	9 (2.82)	—
Instrumentation (frequency [%])	4 (3.05)	1 (4.55)	3 (8.11)	2 (28.57)	10 (3.13)	—
Condensation (frequency [%])	10 (7.63)	2 (9.09)	3 (8.11)	0 (0.00)	15 (4.70)	—
Opening of the pulp chamber and instrumentation (frequency [%])	6 (4.58)	1 (4.55)	3 (8.11)	1 (14.29)	11 (3.45)	—
Opening of the pulp chamber and condensation (frequency [%])	49 (37.40)	8 (36.36)	9 (24.32)	1 (14.29)	67 (21.00)	—
Instrumentation and condensation (frequency [%])	11 (8.40)	0 (0.00)	6 (16.22)	0 (0.00)	17 (5.33)	—
Opening of the pulp chamber, instrumentation, and condensation (frequency [%])	43 (32.82)	9 (40.91)	10 (27.03)	3 (42.86)	65 (20.38)	—

*Note:* Data are presented as the number of students and percentage (%). Chi-square test was used to assess the association between the type of error and nationality. *p*-values < 0.05 were considered statistically significant.

## Data Availability

The data that support the findings of this study are available from the corresponding author upon reasonable request.
